# Enterovirus 71 induces neural cell apoptosis and autophagy through promoting ACOX1 downregulation and ROS generation

**DOI:** 10.1080/21505594.2020.1766790

**Published:** 2020-05-20

**Authors:** Lei You, Junbo Chen, Weiyong Liu, Qi Xiang, Zhen Luo, Wenbiao Wang, Wei Xu, Kailang Wu, Qi Zhang, Yingle Liu, Jianguo Wu

**Affiliations:** a State Key Laboratory of Virology, College of Life Sciences, Wuhan University, Wuhan, China; b Guangzhou Key Laboratory of Virology, Institute of Medical Microbiology, Jinan University, Guangzhou, China

**Keywords:** Hand foot and mouth disease (HFMD), enterovirus 71 (EV71), neurological pathogenesis, peroxisome, acyl-CoA oxidase 1 (ACOX1), reactive oxygen species (ROS)

## Abstract

Enterovirus 71 (EV71) infection causes hand, foot, and mouth disease (HFMD), and even fatal neurological complications. However, the mechanisms underlying EV71 neurological pathogeneses are largely unknown. This study reveals a distinct mechanism by which EV71 induces apoptosis and autophagy in neural cells. EV71 non-structure protein 3D (also known as RNA-dependent RNA polymerase, RdRp) interacts with the peroxisomal protein acyl-CoA oxidase 1 (ACOX1), and contributes to ACOX1 downregulation. Further studies demonstrate that EV71 reduces peroxisome numbers. Additionally, knockdown of ACOX1 or peroxin 19 (PEX19) induces apoptosis and autophagy in neural cells including human neuroblastoma (SK-N-SH) cells and human astrocytoma (U251) cells, and EV71 infection induces neural cell death through attenuating ACOX1 production. Moreover, EV71 infection and ACOX1 knockdown facilitate reactive oxygen species (ROS) production and attenuate the *cytoprotective protein deglycase (DJ-1)/Nuclear factor erythroid 2-related factor 2 (NRF2)/Heme oxygenase 1 (HO-1)* pathway *(DJ-1/NRF2/HO-1)*, which collectively result in ROS accumulation in neural cells. In conclusion, EV71 downregulates ACOX1 protein expression, reduces peroxisome numbers, enhances ROS generation, and attenuates the *DJ-1*/NRF2/HO-1 pathway, thereby inducing apoptosis and autophagy in neural cells. These findings provide new insights into the mechanism underlying EV71-induced neural pathogenesis, and suggest potential treatments for EV71-associated diseases.

## Introduction

Hand, foot, and mouth disease (HFMD) is an infectious disease caused by various enteroviruses, most commonly enterovirus 71 (EV71) [1]. HFMD is normally self-limiting and symptoms usually disappear 7 to 10 days after disease onset. However, a proportion of the patients rapidly develop neurological and systemic complications that can be fatal. Ninety-three percent of the laboratory confirmed deaths of HFMD were associated with EV71 infection [2]. However, the mechanisms of neurological pathogenesis caused by EV71 infection remain unclear and there is no specific antiviral treatment for EV71 infections. EV71 belongs to the *Picornaviridae* family, genus Enterovirus, Enterovirus species A. It is a non-enveloped virus containing a single-stranded, sense-strand, polyadenylated RNA of approximately 7400 nucleotides [3]. The virus genome is initially translated into a single polyprotein, which is promptly cleaved by the viral proteases into 4 structural proteins (VP1–4) and 7 non-structural proteins (2A–C and 3A–D). EV71 3D, containing 462 amino acids, is an RNA-dependent RNA polymerase (RdRp). We previously demonstrated that EV71 3D promotes the activation of the NLRP3 inflammasome through binding to NLRP3 [4]. EV71 infection adopts a number of mechanisms to induce cell death. EV71 2A protease induces apoptotic cell death by cleavage of eIF4G1 [5], 3 C promotes apoptosis through cleaving PinX1 [6], and 2B localized to the mitochondria and induces cell apoptosis by interacting with and activating the proapoptotic protein Bax [7]. Additionally, EV71 triggers neural apoptosis through activation of the Abl-Cdk5 signaling [8], and induces apoptosis and autophagy by regulating miRNAs [9–11].

Peroxisomes are membrane-bound organelle crucial for hydrogen peroxide detoxification. Peroxisomes contain many enzymes that produce or degrade the reactive oxygen species (ROS) and reactive nitrogen species (RNS), they are essential for the maintenance of cellular oxidative balance [12]. In brain, one of the main roles of peroxisomes is to degrade very long chain fatty acids (VLCFA) like C24:0 and C26:0 [13]. Peroxisomal β-oxidation of VLCFAs consists of 4 enzymatic steps, acyl-CoA oxidase 1 (ACOX1) is the first and rate-limiting enzyme. Peroxisome disordered patients lacking peroxisomal functions typically develop severe neurological deficits, ranging from aberrant development of the brain, demyelination and loss of axonal integrity, neuroinflammation, or other neurodegenerative processes [14]. Among several peroxisomal neurodegenerative disorders, the pseudoneonatal adrenoleukodystrophy (p-NALD) is characterized by ACOX1 deficiency, accumulation of VLCFA in tissues, and inflammatory demyelination [15,16]. ACOX1 Deficiency and/or VLCFA accumulation trigger an oxidative stress characterized by ROS overproduction [17]. Similarly, X-linked adrenoleukodystrophy (X-ALD), caused by mutations in a peroxisomal membrane transporter protein ABCD1, is also a disorder of peroxisomal fatty acid β-oxidation, and results in VLCFA accumulation and demyelination [18]. Human autopsy from X-ALD patients showed apoptosis of oligodendrocytes and microglia, which may account for the demyelination process [19,20]. Peroxisomes are also involved in antiviral innate immunity and are primary sites of initiation of type III interferon expression [21]. Besides mitochondria, the RIG-I-like receptor (RLR) adaptor protein, mitochondrial antiviral-signaling protein (MAVS), also locates on peroxisomes [22].

This study reveals a distinct mechanism by which EV71 induces apoptosis and autophagy in neural cells. EV71 downregulates ACOX1 protein expression, reduces peroxisome numbers, and induces neural cell death. Knockdown of ACOX1 or PEX19 leads to the induction of apoptosis and autophagy in neural cells. Additionally, EV71 infection and ACOX1 knockdown result in the promotion of ROS production and attenuation of the anti-oxidative DJ-1/NRF2/HO-1 pathway.

## Materials and methods

### Cells and viruses

Human rhabdomyosarcoma cell line RD, human glioblastoma cell line U251 and human embryonic kidney HEK293 T cells were purchased from the China Center for Type Culture Collection (CCTCC; Wuhan, China), and were cultured in Dulbecco’s modified Eagle’s medium (DMEM; Gibco) supplemented with 10% heat-inactivated fetal calf serum (FBS; Gibco), 100 U/ml penicillin, and 100 μg/ml streptomycin sulfate at 37°C in 5% CO_2_. Human neuroblastoma (SK-N-SH) cells were purchased from CCTCC and cultured in modified Eagle’s medium (MEM, Gibco) supplemented with 10% heat-inactivated FBS, 100 U/ml penicillin, and 100 μg/ml streptomycin sulfate at 37°C in 5% CO_2_. Cells were transfected with Lipofectamine 2000 (Invitrogen) according to the manufacturer’s instructions.

Enterovirus 71 Xiangyang strain (Sub-genotype C4, GenBank accession number JN230523.1) was isolated by our group [23]. Virus propagation and titration were performed by using RD cells, and the 50% tissue culture infectious dose (TCID_50_) was calculated by Reed and Muench method. For EV71 infection, cells were seeded 24–48 h in advance. The cells were rinsed twice with phosphate-buffered saline (PBS), and infected with EV71 at the indicated multiplicity of infection (MOI) in serum-free medium. After adsorption at 37°C for 2 h, the virus-containing medium was removed and the cells were washed twice. Medium containing 2% serum was then added for subsequent incubation.

### Plasmids, small interfering RNAs and reagents

The coding regions of enterovirus 71 3D and human ACOX1 gene were generated by PCR amplification. The coding regions of enterovirus 71 3D were cloned into pCAggs-HA vector, and the coding regions of ACOX1 were cloned into pcDNA3.1(+) vector with 3× Flag tag on the amino terminus. Specific small interfering RNAs (siRNAs) were purchased from Guangzhou RiboBio, the siRNA sequence against human ACOX1 was designed by Guangzhou RiboBio. The siRNA sequence against human PEX19 was as previously reported [24].

Anti-Flag (F1804) and anti-HA (H6908) were purchased from Sigma-Aldrich. Antibodies against β-actin (60008-1-Ig), GAPDH (60004-1-Ig), LC3 (14600-1-AP), p62 (18420-1-AP), DJ-1 (11681-1-AP), NRF2 (16396-1-AP), BACH1 (14018-1-AP), and HO-1 (10701-1-AP) were purchased from Proteintech Group. Rabbit anti-EV71-VP1 (PAB7631-D01P) polyclonal antibody was from Abnova (Taipei city, Taiwan). Anti-PMP70 (ab211533), anti-catalase (ab76110), and anti-ACOX1 (ab184032) were purchased from Abcam. Caspase-3 antibody (#9662) and PARP antibody (#9532) were purchased from Cell Signaling Technology. PEX19 antibody (A5476) was purchased from ABclonal. Dimethyl Sulfoxide, Chloroquine Phosphate (CQ), MG132, N-acetyl-cysteine (NAC), and α-Lipoic acid (LA) were purchased from Sigma-Aldrich. Matchmaker® Gold Yeast Two-Hybrid System (630489) was purchased from TaKaRa. Cell Counting Kit-8 (CCK-8) and Cytotoxicity LDH Assay Kit-WST were purchased from Dojindo Molecular Technologies. FITC Annexin V Apoptosis Detection Kit was purchased from BD Biosciences. Cellular ROS detection assay kit (ab139476) was purchased from Abcam.

### Lentivirus production and infection

Oligonucleotides target human ACOX1 were cloned into pLKO.1, which was a gift from David Root (Addgene plasmid # 10879). Coding sequence of enterovirus 71 3D was inserted into pLenti CMV-3× Flag vector, which was modified from pLenti CMV GFP Puro (a gift from Eric Campeau & Paul Kaufman (Addgene plasmid # 17448)) and contains C-terminal 3 × Flag tag. The plasmids were transfected into HEK293 T cells together with psPAX2 (Addgene #12260) and pMD2.G (Addgene #12259). Culture media was harvested 36 and 60 hours after transfection and filtered through a 0.22 μm filter. RD cells, SK-N-SH cells and U251 cells were infected with the lentiviral particle-containing media in the presence of 8 μg/ml polybrene (Sigma). Culture media was changed 24 hours after infection. After 48 h of culture, 2.5 μg/ml puromycin (Sigma) was added into the media for selection. The knockdown efficiency of the stable cell lines was determined by Western blot.

### Quantitative RT-PCR analysis

Quantitative RT-PCR (qRT-PCR) was used to determine the relative mRNA levels. Total RNA was extracted with TRIzol reagent (Invitrogen) according to the manufacturer’s instructions. RNA was reverse transcribed with oligo(dT) or random primer. Real-time PCR was performed in Light Cycler 480 (Roche) and GAPDH was used as internal control. The following primers were used: EV71-VP1 forward: 5ʹ-AATTGAGTTCCATAGGTG-3ʹ, EV71-VP1 reverse: 5ʹ-CTGTGCGAATTAAGGACAG-3ʹ; ACOX1 forward: 5ʹ-TAACTTCCTCACTCGAAGCCA-3ʹ, ACOX1 reverse: 5ʹ-AGTTCCATGACCCATCTCTGTC-3ʹ; and catalase forward: 5ʹ-AGTGATCGGGGGATTCCAGA-3ʹ, catalase reverse: 5ʹ-GAGGGGTACTTTCCTGTGGC-3ʹ; and PEX19 forward: 5ʹ-CCAAGGATGTGCTGTACCCA-3ʹ, PEX19 reverse: 5ʹ-TTCACCACTGGCACCATCTC-3ʹ; and NRF2 forward: 5ʹ-TCCAGTCAGAAACCAGTGGAT-3ʹ, NRF2 reverse: 5ʹ-GAATGTCTGCGCCAAAAGCTG-3ʹ; and DJ1 forward: 5ʹ-TGCTGGCGTGCGTTCATTTTC-3ʹ, DJ1 reverse: 5ʹ-GGGATGACCGTCTCCATTTCC-3ʹ; and GAPDH forward: 5ʹ-GGAAGGTGAAGGTCGGAGTCAACGG-3ʹ, GAPDH reverse: 5ʹ-CTCGCTCCTGGAAGATGGTGATGGG-3ʹ. Data were normalized to the GAPDH expression level in each sample.

### Western blot analysis and Co-immunoprecipitation

For Western blot analysis, cells were lysed in lysis buffer (50 mM Tris-HCl, pH7.4, 150 mM NaCl, 1% Triton X-100, 1 mM EDTA) in the presence of protease inhibitor mixture (Roche) on ice. Cell lysates (20–60 μg) were separated by 8–12% SDS-PAGE and then transferred to polyvinylidene difluoride (PVDF) membranes (Millipore). The membranes were blocked with 5% skim milk, followed by incubating with specific antibodies. Blots were detected using Clarity Western ECL Substrate (Bio-Rad). For co-immunoprecipitation, cells were lysed in IP lysis buffer (0.025 M Tris-HCl, 0.15 M NaCl, 1 mM EDTA, 1% NP-40, 5% glycerol; pH7.4) in the presence of protease inhibitor mixture on ice. Cell lysates were mixed with specific antibodies or homologous IgG, rocked overnight at 4°C, and incubated with protein G agarose beads (GE Healthcare). Beads were washed and denatured with SDS-PAGE loading buffer and boiling, then analyzed by Western blot. Gray density of Western blots was measured by using ImageJ software (National Institutes of Health, Bethesda, MD).

### Immunoﬂuorescence

Cells were grown in glass-bottom dishes. After transfection and/or EV71 infection, cells were fixed with 4% paraformaldehyde for 15 min, permeabilized with PBS containing 0.2% Triton X-100 for 5 min, washed with PBS, and blocked with 5% bovine serum albumin for 30 min at room temperature. The cells were then incubated with the indicated antibodies at 4°C overnight, followed by incubation with species-specific secondary antibodies labeled with fluorescein for 45 min, and stained with 4ʹ,6-diamidino-2-phenylindole (DAPI). The cells were viewed by fluorescence confocal microscope (Fluoview FV1000, Olympus).

### Apoptosis assay by flow cytometry (FACS)

Apoptosis detection assay was performed by staining cells with annexin V-FITC and propidium iodide (PI) using FITC annexin V apoptosis detection kit (BD Biosciences) according to the manufacturer’s instructions. Briefly, cells were collected with trypsin, washed twice with cold PBS, resuspended in 1X Binding Buffer, and then stained with 5 µl of Annexin-V FITC and 5 µl PI for 15 min at RT (25°C) in the dark. Samples were then analyzed by flow cytometry (Beckman Coulter, CytoFLEX).

### Statistical analysis

The results were presented at the means ± s.d. Student’s t-test was used for comparisons between two groups. P < 0.05 was considered statistically significant, *: P < 0.05, **: P < 0.01, ***: P < 0.001. All statistical analyses were performed with GraphPad Prism 6.0.

## Results

### EV71 3D protein interacts with ACOX1

**Figure 1.**
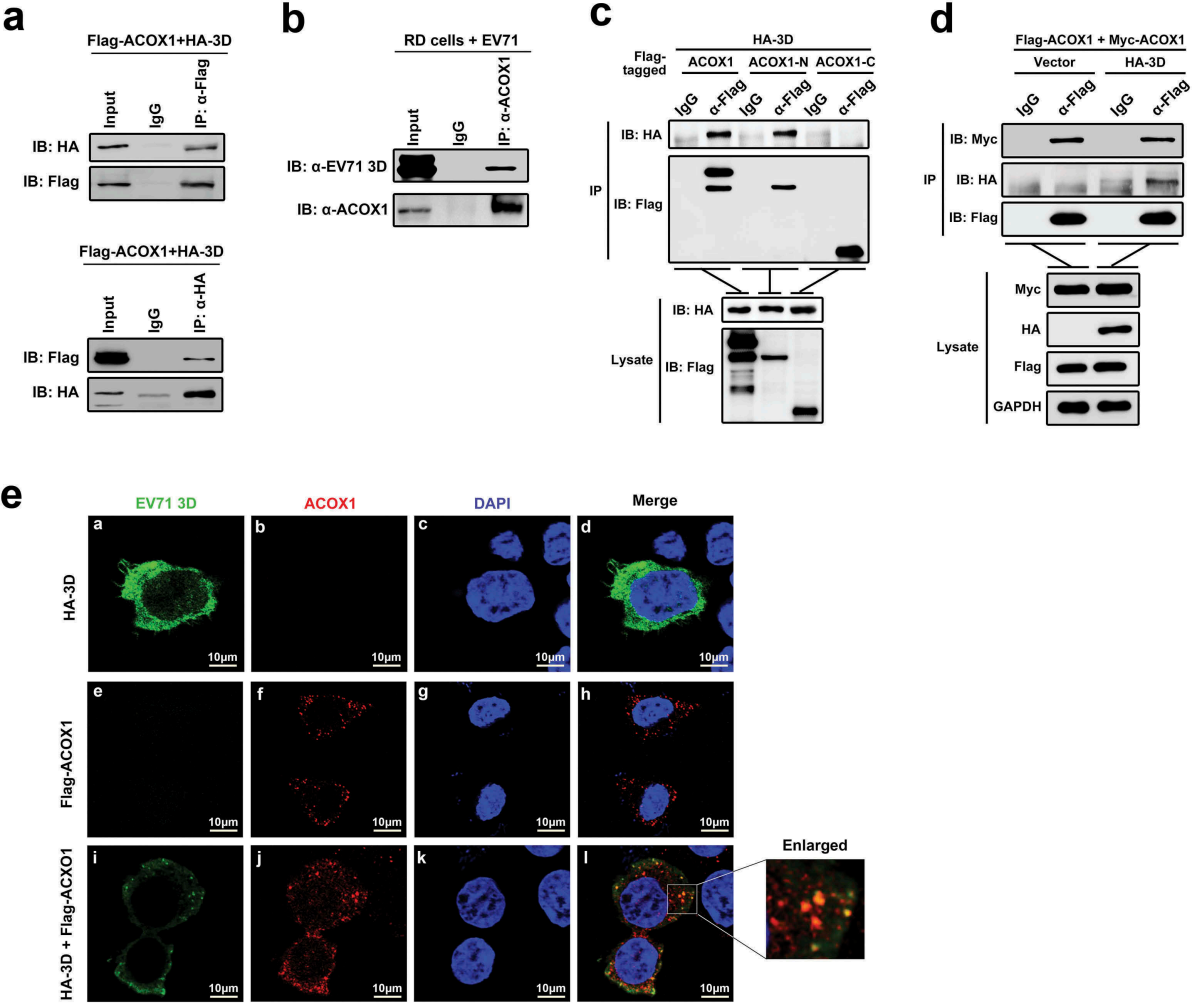
EV71 3D protein interacts with ACOX1. (a) HEK293 T cells were co-transfected with pFlag-ACOX1 expressing Flag-tagged ACOX1 protein and pHA-3D expressing HA-tagged EV71 3D protein. Cell lysates were subjected to immunoprecipitation (IP) with control IgG and anti-Flag antibody (upper panel) or control IgG and anti-HA antibody (lower panel). The immune-precipitates and whole-cell lysates (WCLs) (input) were analyzed by Western blotting with the indicated antibodies. IB, immunoblotting. (b) RD cells were infected with EV71 at an MOI of 2 for 10 hours. Cell lysates were subjected to immunoprecipitation (IP) with control IgG and anti-ACOX1 antibody. The immune-precipitates and whole-cell lysates (WCLs) (input) were analyzed by Western blotting with the indicated antibodies. IB, immunoblotting. (c) HEK293 T cells were co-transfected with plasmid expressing HA-3D and plasmids Flag-ACOX1, Flag-ACOX1-N, or Flag-ACOX1-C expressing Flag-tagged ACOX1, N- or C-terminal ACOX1 components. Cell lysates were immunoprecipitated with IgG or anti-Flag. The immunoprecipitates and lysate were analyzed by Western blotting with the indicated antibodies. IB, immunoblotting. (d) HEK293 T cells were co-transfected with plasmids expressing Flag-ACOX1, Myc-ACOX1 and vector or HA-3D. Cell lysates were immunoprecipitated with IgG or anti-Flag. The immunoprecipitates and lysate were analyzed by Western blotting. (e) U251 cells were transfected with plasmid expressing HA-3D and plasmid expressing Flag-ACOX1. Cells were immunostained with anti-HA and anti-Flag antibodies, and the nucleus was stained with DAPI, cells were analyzed by confocal microscopy.

To determine the role of EV71 3D protein in host–virus interaction, we initially identified host proteins that interact with 3D through yeast two-hybrid screens. Since the full-length 3D protein may exhibit self-activation, we employed a truncated form of 3D (amino acids 95 to 462) as the bait in yeast two-hybrid screens with a universal human normalized cDNA library. The results revealed that 3D (95–462) interacted with the peroxisomal acyl-CoA oxidase 1 (ACOX1). Co-immunoprecipitation (Co-IP) assay confirmed that 3D and ACOX1 interacted with each other in human embryonic kidney (HEK293 T) cells (Figure 1(a)), and 3D protein interact with endogenous ACOX1 in EV71-infected RD cells (Revised Figure 1(b)). The full-length ACOX1 polypeptide (component A) is cleaved proteolytically into the N-terminal 51-kDa (component B) and C-terminal 21-kDa (component C) components in the peroxisome [25], the enzyme exists as a mixture of the forms, A2, B2 C2, and ABC [26]. We generated plasmids expressing the two ACOX1 components, respectively. Co-IP revealed that ACOX1 and the N-terminal component, but not the C-terminal component, were precipitated with 3D (Figure 1(c)), suggesting that the N-terminal domain is required for the binding of ACOX1 to 3D. The two bands in the IP lane of α-Flag-IB Flag correspond in size to the full-length ACOX1 and the N-terminal 51-kDa component of ACOX1. The crystal structure of ACOX1 reveals that ACOX1 acts as a homodimer [27]. To determine whether the interaction of 3D with ACOX1 disrupts ACOX1 homodimerization, HEK293 T cells were co-transfected with plasmids expressing Flag-ACOX1, Myc-ACOX1, and HA-3D. Co-IP showed that Myc-ACOX1 was precipitated with Flag-ACOX1, and such precipitation was not affected by HA-3D (Figure 1(d)), suggesting that 3D had no effect on ACOX1 homodimerization. The colocalization of EV71 3D protein and ACOX1 protein in neural cells was determined by immunofluorescence staining. The results showed that in the presence of Myc-ACOX1, 3D protein formed concentrated dots and partially colocalized with ACOX1 in U251 cells (Figure 1(Ei–l)).

### EV71 downregulates ACOX1 protein expression through 3D

**Figure 2.**
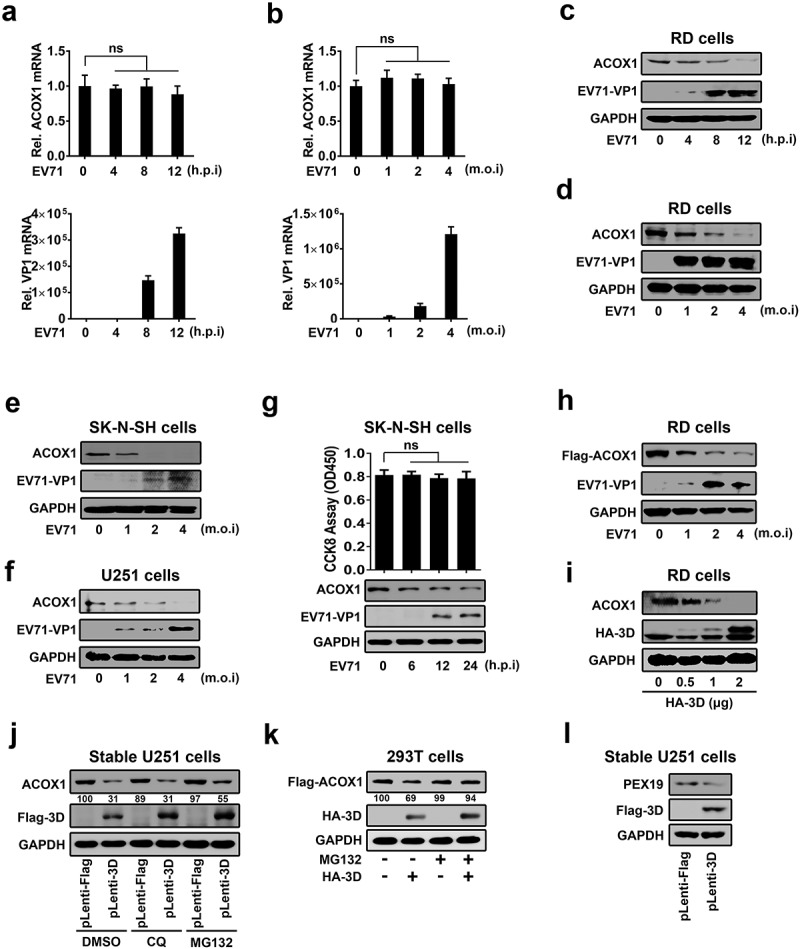
EV71 attenuates ACOX1 protein expression through 3D. (a–d) RD cells were infected with EV71 at an MOI of 2 for different times (a) and (c) or at different MOIs for 12 h (b) and (d). The ACOX1 mRNA (A and B, top panels) and EV71 VP1 mRNA ((a) and (b), bottom panels) were measured by qRT-PCR and normalized to GAPDH mRNA. The ACOX1 and EV71 VP1 proteins in WCLs were determined by Western blot, GAPDH was used as loading control (c) and (d). (e) SK-N-SH cells were infected with EV71 at different MOIs for 48 h. (f) U251 cells were infected with EV71 at different MOIs for 24 h. (g) SK-N-SH cells were infected with EV71 at an MOI of 2 for different times, the viability of cells was measured by CCK8 assay. (h) RD cells were transfected with pFlag-ACOX1 for 24 h, and then infected with EV71 at different MOIs for 12 h. (i) RD cells were transfected with vector or pHA-3D at different concentrations for 36 h. (j) Control U251 cells and stable U251 cells expressing EV71 3D were treated with DMSO, CQ (10 μM), or MG132 (20 μM) for 10 h. (k) HEK293 T cells were co-transfected with pFlag-ACOX1 along with pHA-3D. Cells were treated with or without MG132 (20 μM) for 10 hours before harvest. (l) Control U251 cells and stable U251 cells expressing EV71 3D were analyzed by Western blot.

The effect of EV71 infection on the ACOX1 expression was initially explored in human rhabdomyosarcoma (RD) cells infected with EV71 for different times or at different multiplicity of infection (MOI). Endogenous ACOX1 mRNA was not changed upon EV71 infection (Figure 2(a,b), upper panel). We noticed that EV71 VP1 mRNA was detected (Figure 2(a,b), lower panel), reflecting that EV71 replicates effectively in the cells. In contrast, endogenous ACOX1 protein production was attenuated upon EV71 infection in RD cells in a time-dependent fashion (Figure 2(c)) and a dose-dependent manner (Figure 2(d)). In addition, endogenous ACOX1 protein level was also down-regulated upon EV71 infection in dose-dependent manners in both human neuroblastoma (SK-N-SH) cells (Figure 2(e)) and human astrocytoma (U251) cells (Figure 2(f)). To preclude the effect of cytopathic effect on the expression of ACOX1 protein, cell viability was determined by CCK8 assay in EV71-infected SK-N-SH cells. The results showed that EV71 infection downregulated ACOX1 protein production in the cells without cell death (Figure 2(g)). Moreover, Flag-ACOX1 protein expression was repressed upon EV71 infection in dose-dependent manner in RD cells (Figure 2(h)). Collectively, these results revealed that EV71 infection attenuates the level of ACOX1 protein. Since 3D interacts with ACOX1, the effect of 3D on ACOX1 expression was determined. ACOX1 protein was down-regulated by 3D in RD cells (Figure 2(i)), revealing that EV71 down-regulates ACOX1 through 3D. Furthermore, the pathway by which 3D protein downregulate ACOX1 was explored by the treatments of lysosome inhibitor (chloroquine, CQ) and proteasome inhibitor (MG132). In U251 stable cells, which were infected with Lentivirus-Flag (negative control) or Lentivirus-3D (expressing 3D protein), ACOX1 protein was attenuated by 3D in the presence of DMSO and CQ, but slightly downregulated by 3D in the presence of MG132 (Figure 2(j)). In addition, in HEK293 T cells cotransfected with pFlag-ACOX1 and pHA-3D, 3D protein promotes the downregulation of ACOX1 protein in the presence of DMSO but not MG132 (Figure 2(k)), revealing that proteasome pathway is involved in 3D-mediated ACOX1 degradation. Moreover, peroxisomal protein PEX19 was also down-regulated by 3D protein in U251 stable cells. Taken together, we demonstrate that EV71 infection downregulates ACOX1 protein expression through 3D protein.

### ACOX1 knockdown and EV71 infection attenuate peroxisome numbers

**Figure 3.**
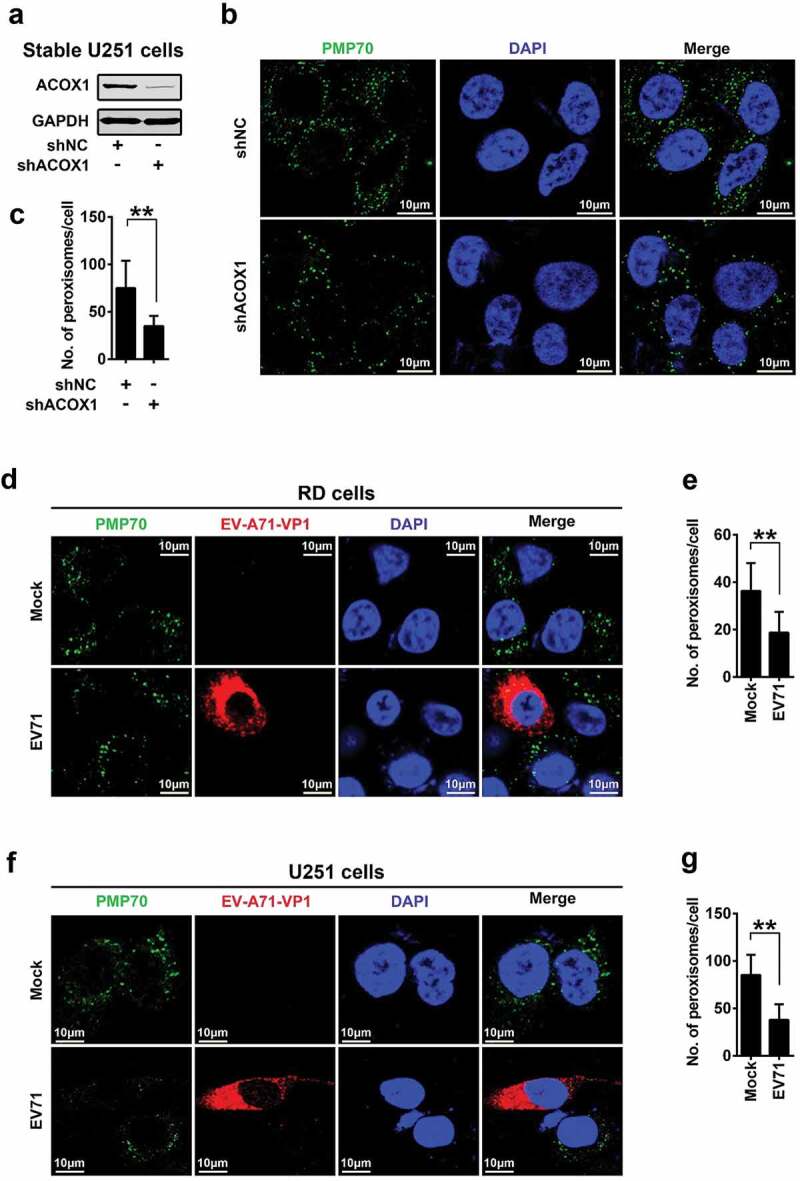
EV71 infection and ACOX1 knockdown reduce peroxisome numbers. (a) Expression of ACOX1 protein in stable U251 cell line was determined by Western blot. (b) Stable shACOX1 U251 cells and control cells were subjected to immunofluorescence microscopy analysis using antibody against PMP70. (c) The numbers of peroxisomes per cell in stable shACOX1 U251 cells (18 cells counted) and control cells (23 cells counted) were analyzed using ImageJ software. (d-g) RD cells (d) and (e) and U251 cells (f) and (g) were mock infected or infected with EV71, cells were immune-stained with anti-PMP70 and anti-EV71 VP1 antibodies (d) and (f). The numbers of peroxisomes per cell in mock infected (11 cells counted) or EV71 infected (10 cells counted) RD cells (e), and in mock infected (14 cells counted) or EV71 infected (11 cells counted) U251 cells (g) were analyzed using ImageJ software.

**Figure 4.**
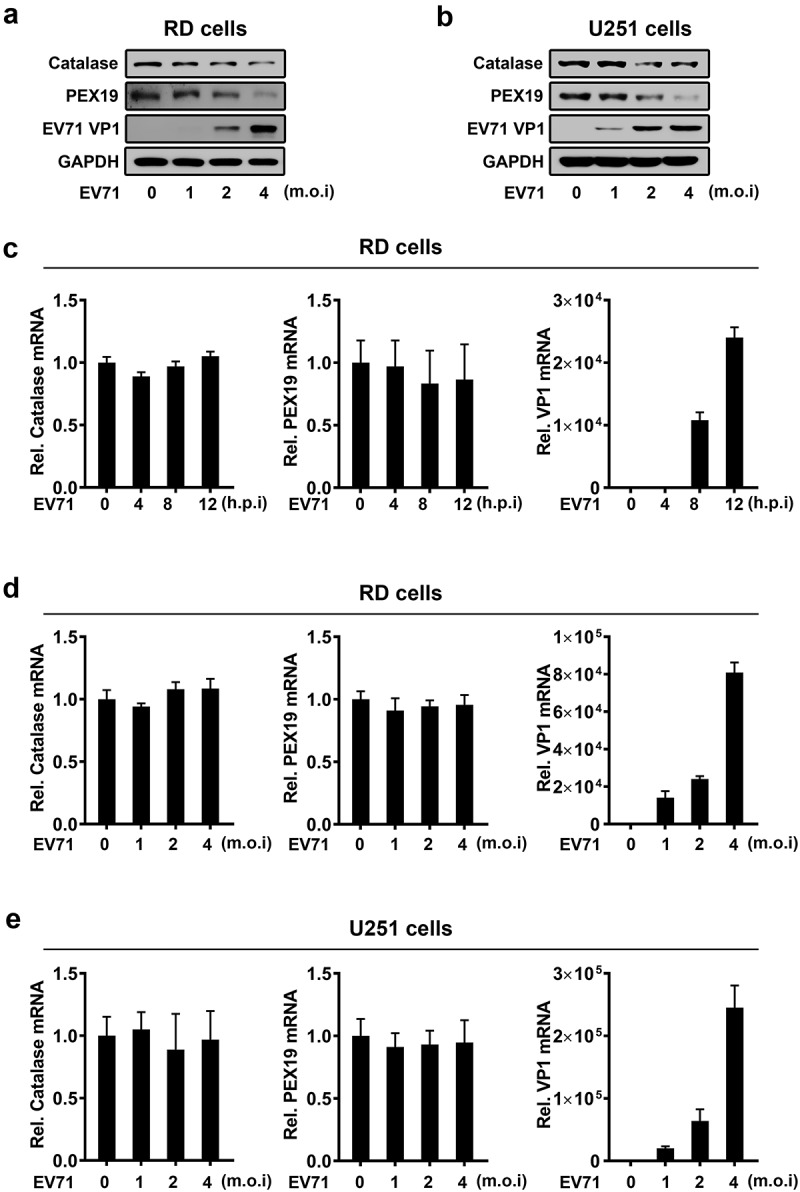
EV71 infection down-regulates expression of peroxisomal proteins. (a) and (b) RD cells (a) and U251 cells (b) were infected with EV71 at the indicated MOIs for 12 hours (A) and 24 hours (B), protein levels were determined by Western blot. (c) and (d) RD cells were infected with EV71 at a MOI of 2 for different times (c) or at different MOIs for 12 h (d). (e) U251 cells were infected with EV71 at different MOIs for 24 h. The mRNA levels of catalase, PEX19, and EV71 VP1 were measured by qRT-PCR and normalized to GAPDH mRNA.

Analysis of skin fibroblasts showed that the numbers of peroxisomes were decreased in patients with peroxisomal ACOX1 deficiency [15,16]. To validate the effect of ACOX1 knockdown on the numbers of peroxisomes in neural cells, we applied shRNA specifically targeting ACOX1 (shACOX1) to generate stable ACOX1-knockdown U251 cell line (Figure 3(a)). Peroxisomes in the stable U251 cells were visualized under immunofluorescence microscopy using antibodies against peroxisomal protein PMP70 as described previously [28]. The numbers of peroxisomes were significantly reduced by shACOX1 in stable U251 cells (Figure 3(b,c)), indicating that ACOX1 knockdown down-regulates peroxisomes. The infections of Flavivirus and human immunodeficiency virus type 1 (HIV-1) lead to attenuating peroxisome biogenesis [24,29]. Here, we explored the effect of EV71 infection on peroxisome numbers. The numbers of peroxisomes were decreased in EV71-infected RD cells (Figure 3(d,e)) and also down-regulated in EV71-infected U251 cells (Figure 3(f,g)), suggesting that EV71 infection reduces peroxisome numbers. Protein levels of peroxisomal proteins such as catalase and PEX19 were also reduced upon EV71 infection in RD cells and U251 cells (Figure 4(a,b)), while the mRNA levels of catalase and PEX19 were not affected upon EV71 infection (Figure 4(c-e)). Collectively, these results reveal that ACOX1 knockdown and EV71 infection reduce peroxisome numbers.

### ACOX1 knockdown and peroxisome disruption promote apoptosis and autophagy in neural cells

**Figure 5.**
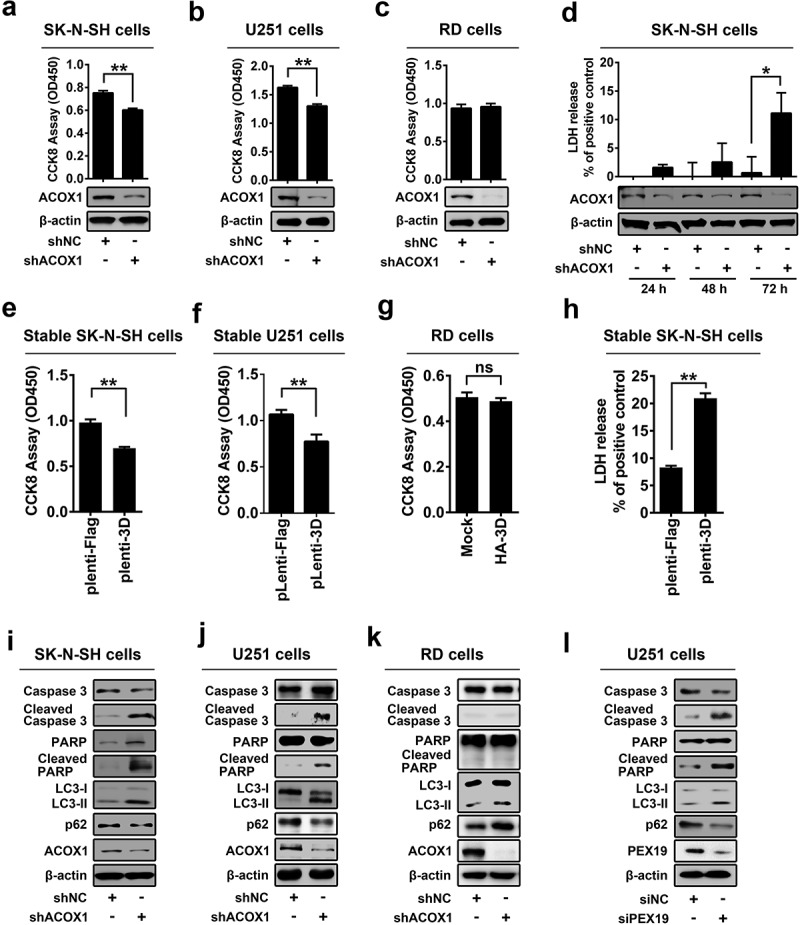
ACOX1 knockdown and peroxisome disruption promotes apoptosis and autophagy in neural cells. (a–c) SK-N-SH cells were transfected with negative control shRNA (shNC) or shACOX1 for 72 h (a). U251 cells were transfected with shNC or shACOX1 for 60 h (b). RD cells were transfected with negative control shRNA (shNC) or shACOX1 for 72 h (c). The viability of cells was measured by CCK8 assay. The expression of ACOX1 protein was determined by Western blot. (d) SK-N-SH cells were transfected with shNC or sh-ACOX1 at different times. LDH activity in cell culture supernatants was tested. The expression of ACOX1 protein was determined by Western blot. (e) The viability of control SK-N-SH cells and stable SK-N-SH cells expressing EV71 3D was measured by CCK8 assay. (f) The viability of control U251 cells and stable U251 cells expressing EV71 3D was measured by CCK8 assay. (g) The viability of RD cells transfected with or without pHA-3D was measured by CCK8 assay. (h) The viability of control SK-N-SH cells and stable SK-N-SH cells expressing EV71 3D was measured by LDH assay. (i–k) SK-N-SH cells (i), U251 cells (j), and RD cells (k) were transfected with shNC or shACOX1 for 72 h. Protein levels in treated cells were determined by Western blot analyses using the corresponding antibodies as indicated. (l) U251 cells were transfected with negative control siRNA (siNC) or siPEX19 for 36 h. Cell lysates were analyzed by Western blot.

Mutations in adrenoleukodystrophy protein (ALDP) are associated with apoptosis of oligodendrocytes and microglia in patient tissues [19,20]. We speculated that ACOX1 knockdown may cause programmed cell death. Cell Counting Kit-8 (CCK-8) assays showed that cell viability was decreased by shACOX1 in SK-N-SH cells (Figure 5(a)) and U251 cells (Figure 5(b)), but relatively unaffected by shACOX1 in RD cells (Figure 5(c)), suggesting that ACOX knockdown attenuates viability of neural cells (SK-N-SH cells and U251 cells), but not non-neural cells (RD cells). We noticed that ACOX1 protein was attenuated by shACOX1 in the three cell lines (Figure 5(a-c)), confirming that shACOX1 is effective. To further clarify the effects of ACOX1 knockdown on cell proliferation and cell cytotoxicity, we measured the activity of lactate dehydrogenase (LDH), which is released from damaged cells. The level of released LDH in the supernatant was promoted by shACOX1 (Figure 5(d)), indicating that ACOX1 knockdown promotes SK-N-SH cell damage. Additionally, cell viability was significantly reduced in Lentivirus-3D stable SK-N-SH cells (Figure 5(e)), and Lentivirus-3D stable U251 cells (Figure 5(f)), but not in HA-3D transfected RD cells (Figure 5(g)). In addition, LDH measurement showed that cell cytotoxicity increased in Lentivirus-3D stable SK-N-SH cells (Figure 5(h)). These results implicating that shACOX1 and 3D down-regulates cell viability of neural cells SK-N-SH and U251 but not non-neural RD cells.

Moreover, the effects of ACOX1 knockdown on apoptosis and autophagy were determined in SK-N-SH, U251, and RD cells transfected with shNC or shACOX1. The Caspase-3 and poly(ADP-ribose) polymerase (PARP), two molecular indicators of cellular apoptosis, as well as the microtubule-associated protein 1 light chain 3 (LC3) and sequestosome 1 (p62/SQSTM1), two molecular indicators of autophagy, were evaluated. The levels of cleaved Casp-3 and cleaved PARP were promoted by shACOX1 in SK-N-SH cells (Figure 5(i)), indicating that ACOX1 knockdown induces apoptosis in SK-N-SH cells. Conversion of LC3-I to LC3-II by the phosphatidylethanolamine is considered a marker of autophagosome formation and accumulation [30]. The adapter protein p62/SQSTM1 mediates delivery of ubiquitinated cargo to autophagosomes, and it is degraded along with its cargo, accumulation of p62 indicates disrupted autophagic degradation [31]. Here, we showed that LC3-II level was facilitated, while p62/SQSTM1 level was attenuated, by shACOX1 in SK-N-SH cells (Figure 5(i)). Similarly, cleaved Casp-3, cleaved PARP and LC3-II level were up-regulated by shACOX1 in U251 cells, while p62 was down-regulated (Figure 5(j)), suggesting that ACOX1 knockdown leads to the induction of apoptosis and autophagy in neural cells SK-N-SH and U251. In contrast, cleaved Casp-3, cleaved PARP were relatively unchanged, while p62/SQSTM1, and LC3-II were increased by shACOX1 in RD cells (Figure 5(k)), indicating that ACOX1 knockdown displays no effect on apoptosis, but disrupts the autophagy pathway in RD cells. The effect of peroxin 19 (PEX19), a protein essential for peroxisome membrane assembly and maintenance [32], on the regulation of programmed cell death was also determined. In U251 cells, cleaved Casp-3, cleaved PARP, and LC3-II were promoted by siPEX19, while p62/SQSTM1 was attenuated by siPEX19 (Figure 5(l)), indicating that PEX19 knockdown leads to the induction of apoptosis and autophagy. Taken together, we demonstrate that ACOX1 knockdown and peroxisome disruption promotes apoptosis and autophagy in neural cells.

### EV71 induces neural cell death through attenuating ACOX1 production

Since ACOX1 is down-regulated upon EV71 infection, and ACOX1 knockdown promotes neural cell apoptosis and autophagy, we determined whether EV71 induces neural cell death through the downregulation of ACOX1. We firstly determined the effect of ACOX1 knockdown and overexpression on the replication of EV71. EV71 VP1 protein and 3D protein productions (Figure S1(a)) and EV71 VP1 mRNA expression (Figure S1(b)) were enhanced by siACOX1 transfected RD cells. Similarly, EV71 VP1 protein was promoted in shACOX1 stable RD cells (Figure S1(c)) and stable U251 cells (Figure S1(d)). In contrast, the production of EV71 VP1 protein was attenuated by overexpressed ACOX1 in RD cells (Figure S1(e,f)). We cannot draw the conclusion that EV71 induces neural cell death through overexpressing ACOX1, as the replication of EV71 is attenuated by ACOX1 overexpression, and the decrease of EV71 replication itself results in less cell death. We further sought to determine the role of ACOX1 in EV71-induced cell death through attenuating ACOX1 production by using shACOX1.

**Figure 6.**
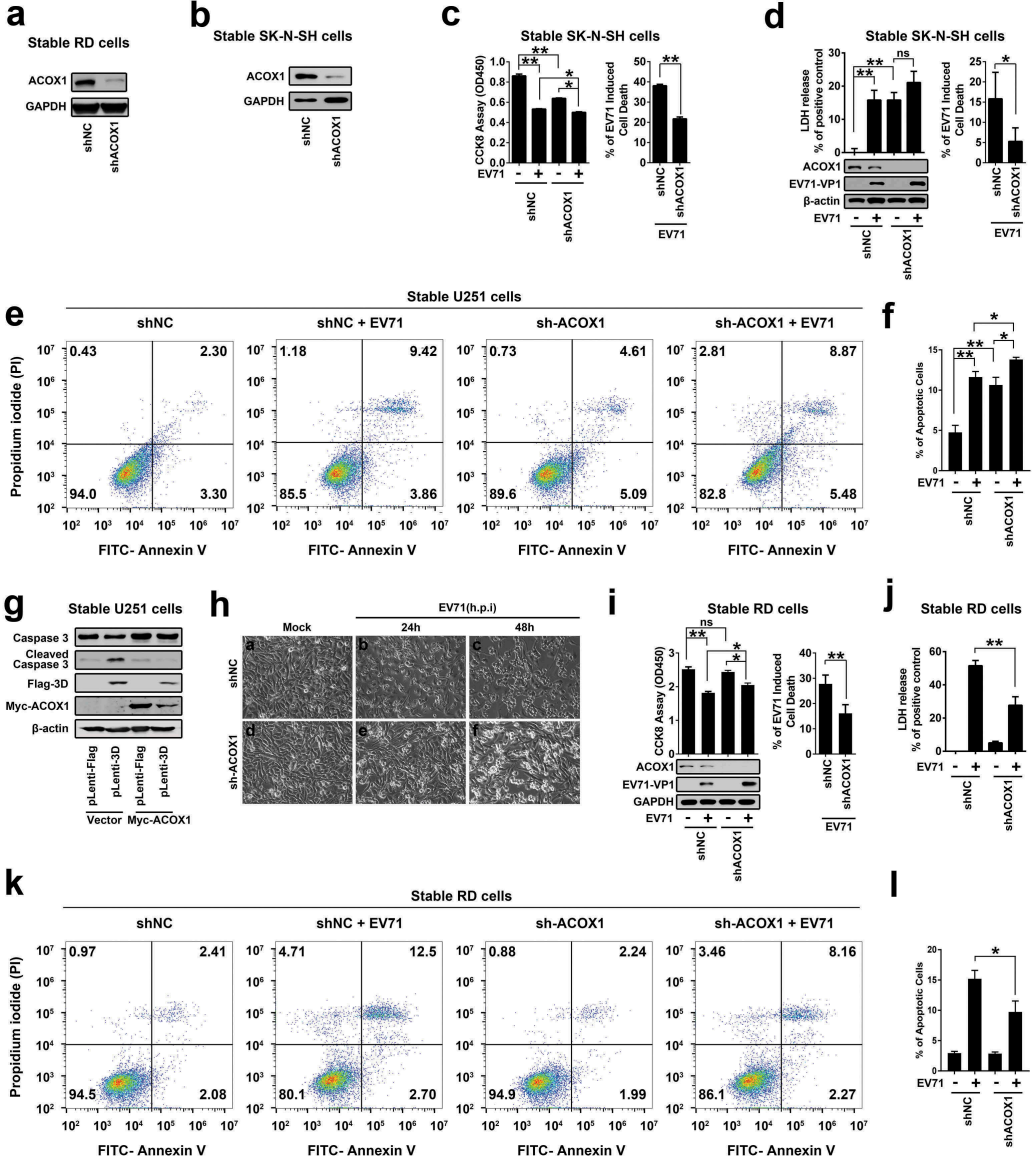
EV71 induces neural cell death through attenuating ACOX1 production. (a) and (b) Expression of ACOX1 protein in ACOX1 knockdown stable RD cells (a) and stable SK-N-SH cells (b) were determined by Western blot. (c-f) shNC or shACOX1 stable SK-N-SH cells were mock-infected or infected with EV71 (MOI = 5). Cell viability was analyzed by CCK8 assay at 72 h after infection ((c), left panel), and cell death induced by EV71 infection was calculated as percentage of the CCK8 value reduction of shNC or shACOX1 stable cells infected by EV71 ((c), right panel). LDH activity in cell culture supernatants was measured at 48 h post-infection, expression of EV71 VP1 and ACOX1 were determined by Western blot ((d), left panel), and cell death induced by EV71 infection was calculated as percentage of LDH release from shNC or shACOX1 stable cells infected by EV71 ((d), right panel). Cell apoptosis was analyzed by annexin V-FITC and propidium iodide (PI) staining and flow cytometry at 36 h post-infection (e) and (f). Percentage of apoptotic cells in three independent experiments were calculated (f). (g) Control U251 cells and stable U251 cells expressing EV71 3D were transfected with vector or plasmid expressing Myc-ACOX1; 48 hours later, expression of proteins was determined by Western blot as indicated. (h) shNC or shACOX1 stable RD cells were mock infected or infected with EV71 (MOI = 0.5). Photographs were taken at 24 or 48 h post-infection. (i–l) shNC or shACOX1 stable RD cells were mock infected or infected with EV71 (MOI = 0.25). Cell viability was analyzed by CCK8 assay at 24 h post-infection, expression of EV71 VP1 and ACOX1 were determined by Western blot ((i), left panel), and cell death induced by EV71 infection was calculated as percentage of the CCK8 value reduction of shNC or shACOX1 stable cells infected by EV71 ((i), right panel). LDH activity in cell culture supernatants was measured (j). Cell apoptosis was analyzed by annexin V-FITC and propidium iodide (PI) staining and flow cytometry at 12 h post-infection (k) and (l). Percentage of apoptotic cells in three independent experiments was calculated (l).

RD cells and SK-N-SH cells stably expressed shRNA targeting ACOX1 (shACOX1) or its negative control (shNC) were generated (Figure 6(a,b)). The cytopathogenic effect (CPE) induced upon EV71 infection in shACOX1 stable SK-N-SH cells and U251 cells were evaluated by three different approaches. The level of cell cytotoxicity was measured by cell counting Kit-8 (CCK-8) assay (Figure 6(c)) and lactate dehydrogenase (LDH) release (Figure 6(d)), and the percentage of cell apoptosis was analyzed by annexin V-FITC and propidium iodide (PI) staining and flow cytometry (Figure 6(e,f)). The results showed that the level of cell cytotoxicity and cell apoptosis was induced by EV71 infection, shACOX1 and shACOX1 plus EV71 infection (Figure 6(c) left panel, Figure 6(d) left panel, and Figure 6(e,f)), but EV71-mediated induction was attenuated in the presence of shACOX1 (Figure 6(c) right panel, Figure 6(d) right panel, and Figure 6(e,f)). Moreover, in stable U251 cells, the level of cleaved Casp-3 was induced by the expression of 3D protein, and 3D-induced increase of cleaved Casp-3 level was attenuated by expression of ACOX1 protein (Figure 6(g)). Collectively, these results reveal that ACOX1 knock-down attenuates EV71-induced CPE and cell death in neural cells.

Since knockdown of ACOX1 disrupts the autophagy pathway in RD cells, we further explored the effect of ACOX1 knockdown on the regulation of EV71-induced CPE in RD cells. We noticed that stable knockdown of ACOX1 had no effect on the morphology of RD cells (Figure 6 Ha *vs*. Hd). EV71-induced CPE in shACOX1 stable RD cells (Figure 6 He and Hf *vs*. Hd) was lower than that in shNC stable RD cells (Figure 6 Hc and Hb *vs*. Ha). Similarly, CCK-8 assays showed that the effect of EV71 infection on cell proliferation (Figure 6(i), left) and CPE or cell death (Figure 6(i), right) were reduced by shACOX1 in stable RD cells. Moreover, LDH measurements showed that EV71-induced LDH release was down-regulated by shACOX1 in stable RD cells (Figure 6(j)). Annexin V-FITC and PI staining showed that EV71-induced cell apoptosis was reduced by shACOX1 in stable RD cells (Figure 6(k,l)). Therefore, these results suggest that EV71 induces cell death, and ACOX1 knock-down alleviates EV71-induced cell death in non-neural RD cells.

### EV71 infection and ACOX1 knockdown promote neural cell death through inducing ROS and attenuating the DJ-1/NRF2/HO-1 pathway

**Figure 7.**
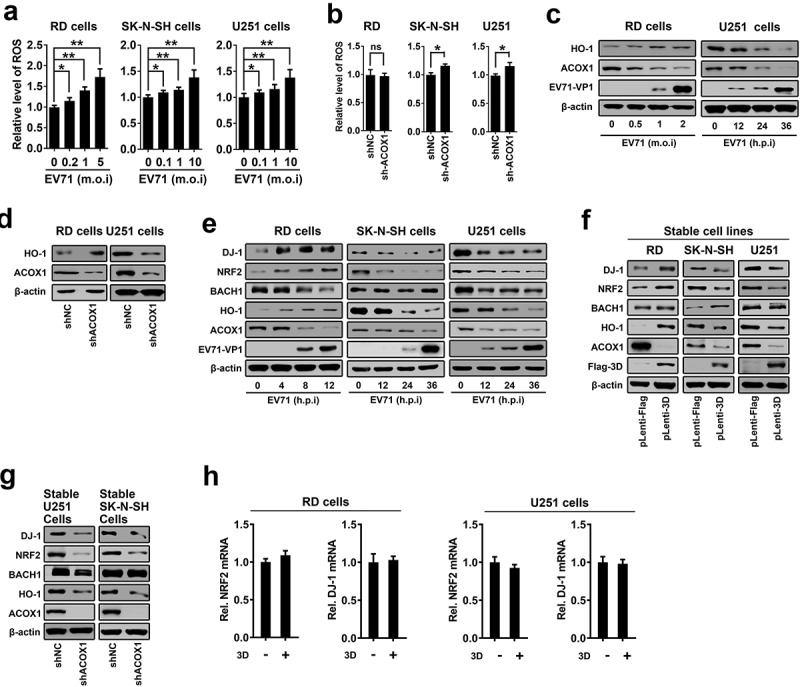
EV71 infection and ACOX1 knockdown promote neural cell death through inducing ROS and attenuating the DJ-1/NRF2/HO-1 pathway. (a) RD cells were infected with EV71 at the indicated MOIs for 18 h. SK-N-SH cells were infected with EV71 at the indicated MOIs for 48 h. U251 cells were infected with EV71 at the indicated MOIs for 24 h. Cellular ROS level was tested using a ROS detection kit. (b) Cellular ROS levels in stable ACOX1 knockdown RD cells, stable ACOX1 knockdown SK-N-SH cells, and stable ACOX1 knockdown U251 cells were tested. (c) RD cells were infected with EV71 at different MOIs for 12 h. U251 cells were infected with EV71 at an MOI of 2 for different times. (d) Stable ACOX1 knockdown cells were subjected to Western blot analysis. (e) RD cells were infected with EV71 at an MOI of 2 for different times. SK-N-SH cells were infected with EV71 at an MOI of 2 for different times. U251 cells were infected with EV71 at an MOI of 2 for different times. (f) Stable EV71 3D expressing cells were subjected to Western blot analysis. (g) Stable ACOX1 knockdown cells were subjected to Western blot analysis. (h) RD cells, and U251 cells were transfected with vector or plasmid expressing EV71 3D protein, 48 hours after transfection, the mRNA levels of NRF2, and DJ-1 were measured by qRT-PCR and normalized to GAPDH mRNA.

Very-long-chain fatty acid (VLCFA) accumulation and ACOX1 knockdown facilitate reactive oxygen species (ROS) production in neural oligodendrocytes [17]. ROS overproduction contributes to the modification of mitochondrial activity and the trigger of cell death [33]. We speculated that EV71 infection may cause cell death through inducing ROS. Notably, ROS production was induced upon EV71 infection in RD cells, SK-N-SH cells, and U251 cells (Figure 7(a)). Interestingly, knockdown of ACOX1 had no effect on ROS production in non-neural RD cells (Figure 7(b), left panel), but promoted ROS production in neuroblastoma SK-N-SH cells, and astrocytoma U251 cells (Figure 7(b), middle and right panels).

Heme oxygenase 1 (HO-1) is the inducible isoform of the first and rate-limiting enzyme of heme degradation, and induced by many physiological and pathological stimuli including oxidative stress, cytokines, bacterial compounds, and growth factors [34]. Induction of HO-1 protects cells against the cytotoxicity of oxidative stress and apoptotic cell death. Here, the effects of EV71 infection and ACOX1 knockdown on the expression of HO-1 were investigated. Notably, HO-1 production was promoted upon EV71 infection in RD cells (Figure 7(c), left panel), and in contrast, HO-1 production was attenuated upon EV71 infection in U251 cells (Figure 7(c), right panel), while ACOX1 generation was down-regulated upon EV71 infection in both RD cells and U251 cells (Figure 7(c)). Interestingly, ACOX1 knockdown promoted HO-1 production in RD cells (Figure 7(d), left panel), but attenuated HO-1 production in U251 cells (Figure 7(d), right panel). Therefore, these results reveal that EV71 infection and ACOX1 knockdown facilitate antioxidant protein HO-1 production in non-neural RD cells, but attenuate HO-1 production in astrocytoma U251 cells.

The mechanisms by which EV71 infection and ACOX1 knockdown regulate HO-1 production were further revealed. Human nuclear factor erythroid 2-related factor 2 (NRF2) is involved in the cellular protection against ROS through antioxidant response element (ARE)-directed induction of several antioxidant enzymes, including HO-1 [35]. Deglycase (DJ-1, also known as Parkinson disease protein 7, PARK7) stabilizes NRF2 to protect against oxidative stress [36]). BRCA1-associated C-terminal helicase 1 (BACH-1, also known as BRCA1-interacting protein 1, BRIP1) represses HO-1 transcription by binding to HO-1 promoter [37]. Here, we showed that EV71 infection facilitated DJ-1, NRF2, and HO-1 production and attenuated BACH-1 and ACOX1 production in RD cells (Figure 7(e), left panel), but repressed DJ-1, NRF2, HO-1, and ACOX1 production in SK-N-SH cells (Figure 7(e), middle panel), and repressed DJ-1, NRF2, BACH-1, HO-1, and ACOX1 production in U251 cells (Figure 7(e), right panel). Additionally, DJ-1, NRF2, and HO-1 were up-regulated by EV71 3D in stably RD cells (Figure 7(f), left panel); while DJ-1, NRF2, HO-1, and ACOX1, but not BACH-1, were down-regulated by EV71 3D in stably SK-N-SH cells and U251 cells (Figure 7(f), middle and right panels). Moreover, DJ-1, NRF2, and HO-1, but not BACH-1, were attenuated by stably expressed shACOX1 in U251 cells (Figure 7(g), left) and SK-N-SH cells (Figure 7(g), right). The mRNA levels of NRF2 and DJ-1 were not affected by 3D overproduction in RD cells, and U251 cells (Figure 7(h)). Therefore, these data suggest that EV71 infection, 3D over-expression, and ACOX1 knockdown attenuate anti-oxidative protein HO-1 production in neural cells through attenuating the DJ-1/NRF2/HO-1 pathway.

**Figure 8.**
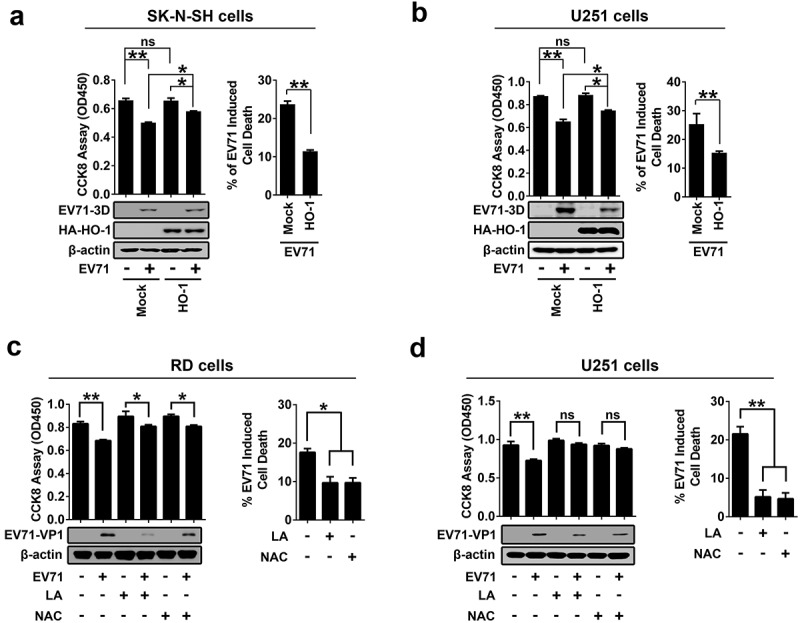
EV71 infection-induced cell death can be rescued by antioxidants treatment. (a) and (b): SK-N-SH cells (a) and U251 cells (b) were transfected with or without pHA-HO-1, 24 hours after transfection, cells were infected with EV71 at an MOI of 2 for 24 h. (c) and (d) RD cells (c) or U251 cells (d) were infected with EV71 at an MOI of 0.25 or at an MOI of 2, respectively, for 24 h. LA (1 mM) or NAC (0.4 mM) were added into cell culture supernatant at 2 h post-infection. (a-d) Treated cells were analyzed using CCK-8 assay kit and Western blot ((a-d), left panels), percentages of EV71 infection-induced cell death with or without HO-1 transfection or antioxidants treatment were calculated ((a-d), right panels).

The role of EV71-induced ROS production in the regulation of cell death was further illuminated. EV71-induced cell death was suppressed by the antioxidant defense protein HO-1 in SK-N-SH cells (Figure 8(a)) and in U251 cells (Figure 8(b)), and by the antioxidants N-acetyl-cysteine (NAC) or α-lipoic acid (LA) in RD cells (Figure 8(c)) and U251 cells (Figure 8(d)). We noticed that EV71 replication, as indicated by VP1 production, was attenuated by NAC and LA in RD and U251 cells (Figure 8(c,d)). Taken together, our results demonstrate that EV71 infection and ACOX1 knockdown lead to the induction of neural cell death by inducing ROS production and attenuating the DJ-1/NRF2/HO-1 pathway. The results also suggest that downregulation of antioxidative protein HO-1 results in the increased sensibility of neural cells to oxidative stress and cell death induced by EV71 infection and ACOX1 knockdown.

**Figure 9.**
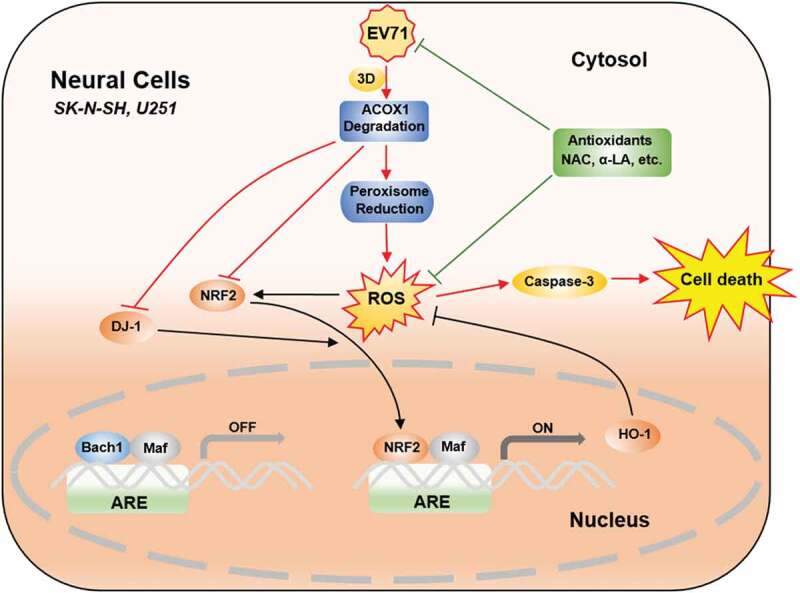
EV71 induces neural cell apoptosis and autophagy through promoting ACOX1 downregulation and ROS accumulation. In normal conditions, antioxidant response elements (AREs) are bound by small Maf dimers or other repressive factors like Bach1. Reactive oxidative stress (ROS) causes the nuclear translocation of NRF2. The activation of NRF2 is further promoted by positive pathway modulators like DJ-1. As a result, antioxidant response genes, like HO-1, are transcriptionally induced, and oxidative damage is minimized. In neural cells, including SK-N-SH cells and U251 cells, EV71 infection results in ACOX1 downregulation through 3D protein, further leads to peroxisome reduction. ACOX1 and peroxisome reduction increase ROS generation. ACOX1 knockdown downregulates DJ-1, NRF2, and HO-1 expression. ROS generation increase and antioxidant response downregulation together result in ROS accumulation, which further leads to caspase-3 activation and cell death. Treatment of antioxidants, including NAC and α-LA, inhibits EV71 replication, scavenges ROS production, and protects neural cells from death.

## Discussion

EV71 infection commonly causes HFMD, which is normally self-limiting, however, a proportion of HFMD patients rapidly develops fatal neurological and systemic complications [2], and EV71 had been isolated from brainstem and spinal cords of fetal cases of human infection [38–40]. Neurogenic pulmonary edema (NPE) is the main cause of death, with neural degeneration and perivascular lymphocytic inflammation in the brainstem [41]. However, the mechanisms underlying neurological pathogeneses of EV71 infection remain largely unclear. This study reveals a distinct mechanism by which EV71 induces neural cell death through promoting ACOX1 downregulation and ROS accumulation.

Infections of several viruses had been reported to be associated with peroxisome dysregulation. The infections of Flaviviruses, including West Nile virus (WNV) and Dengue virus (DENV), result in the reduction of peroxisomes through capsid protein-mediated sequestration and degradation of PEX19 [24]. Human immunodeficiency virus type 1 (HIV-1) infection attenuates peroxisomes by upregulation of miRNAs targeting peroxisome biogenesis factors, and peroxisome disorder is associated with inherited neurodegeneration and neurocognitive disorder in chronic HIV-1 infection [29]. Here, for the first time, we show that EV71 disturbs peroxisomal function by targeting its critical protein ACOX1, and reveal that peroxisome dysfunction contributes to the neural pathogenesis of EV71 infection. Given the roles of peroxisomes in nervous system function and antiviral defense, they might involve in pathological mechanisms underlie multiple viral diseases.

EV71 3D is the viral RNA-dependent RNA polymerase (RdRp) and plays an essential role in viral negative-strand RNA synthesis [42,43]. 3D also regulates cell S-phase arrest and antagonizes type II interferon (IFN-γ) antiviral activity [44,45]. We recently revealed that EV71 3D facilitates the NLRP3 inflammasome activation [4]. However, the effect of 3D on the regulation of EV71 neurological pathogeneses has not been reported until this study. We reveal that 3D interacts with ACOX1, attenuates ACOX1 production, and promotes neural cell death. We found that EV71 3D partially colocalized with ACOX1 in peroxisomes in neural cells. It was reported that co-factor-bound and oligomeric proteins can be transported into peroxisomes [46]; thus, we speculate that EV71 3D might be transported into peroxisomes along with ACOX1. 3D generally shares structure/sequence similarity with homologous RdRp from poliovirus, coxsackievirus, and rhinovirus [47]. RdRp of other viruses might also have similar functions, which needs further studies.

Two studies have reported EV71-induced ROS generation: firstly, EV71 induces ROS formation through integrin beta1/EGFR-Rac1-dependent oxidative stress [48], and secondly, EV71 induces mitochondrial ROS generation [49]. ACOX1 deficiency leads to VLCFA accumulation, which triggers an oxidative stress with ROS overproduction [17]. Peroxisome is a key player in the dynamic spin of ROS metabolism and oxidative injury [50,51]. Disturbances in peroxisomal function sensitize cells to oxidative stress, and cultured cerebellar neurons from peroxisome-deficient mice display increased oxidative stress and apoptosis [52]. We reveal that EV71 induces ROS accumulation through attenuating ACOX1 production and peroxisome biogenesis, thereby causing oxidative stress and cell injury.

HO-1 is an inducible and detoxifying enzyme critical for limiting oxidative stress, inflammation, and cellular injury within the central nervous system (CNS) and other tissues [53]. HO-1 deficiency in the brains of HIV-1 infected individuals is correlated with cognitive dysfunction, HIV-1 replication in the CNS, and neuroimmune activation [54]. EV71 infection and ACOX1 knockdown induce ROS production and activate antioxidative the DJ-1/NRF2/HO-1 pathway in RD cells, however, result in the reduction of HO-1 in neural cells. In accordant with these findings, it is known that the brain and nervous system are inadequately equipped with antioxidant defense systems and are prone to oxidative stress [55]. HO-1 suppresses the replication of multiple viruses, including Ebola virus (EBoV) [56], Dengue virus (DENV) [57], Respiratory syncytial virus (RSV) [58], EV71 [48], as well as Hepatitis C virus (HCV), Hepatitis B virus (HBV), and HIV-1 [59]. The down-regulation of HO-1 by EV71 in neural cells not only results in the loss of its cytoprotective effect, but also permits virus replication, suggesting that HO-1 reduction contributes to EV71-mediated neurodegeneration.

It was reported that EV71 induces cell apoptosis through the mitochondrial pathway mediated by Caspase 9 [60,61], viral protein synthesis is essential for the induction of apoptosis in human glioblastoma SF268 cells [62], and EV71 3 C triggers apoptosis in SF268 cells [63]. This study revealed a distinct mechanism by which EV71 induces apoptosis and autophagy through attenuation of ACOX1 production and promotion of ROS generation in neuroblastoma and astrocytoma cells. ACOX1-knockdown reduced EV71-induced cell death by 45–68% in SK-N-SH cells, and antioxidant treatment attenuate EV71-induced cell death by 75% in U251 cells. These results suggested that ACOX1 attenuation and ROS generation play major roles in EV71-induced cell death in neural cells. In contrast, in non-neural RD cells, ACOX1 knockdown displays no apparent effect on apoptosis and autophagy. ACOX1 seems to be essential in neural cells as ACOX1 knockdown represses the antioxidative DJ-1/NRF2/HO-1 pathway, attenuates HO-1 production, and enhances ROS generation and accumulation in these cells. However, ACOX1 knockdown facilitates HO-1 production without ROS accumulation in non-neural RD cells.

Connectively, this study reveals that EV71 reduces ACOX1 protein expression, down-regulates peroxisome numbers, and enhances ROS generation and accumulation, thereby inducing apoptosis and autophagy in neural cells (Figure 9). The data provide insights into the mechanism underlying EV71-induced neural pathogenesis, and suggest potential treatments.
