# Supplementary figures and images for: Enterovirus 71 induces neural cell apoptosis and autophagy through promoting ACOX1 downregulation and ROS generation
Source: Virulence. 2020 May 20;11(1):537–53. doi: 10.1080/21505594.2020.1766790 (PMC7250321; doi:10.1080/21505594.2020.1766790)

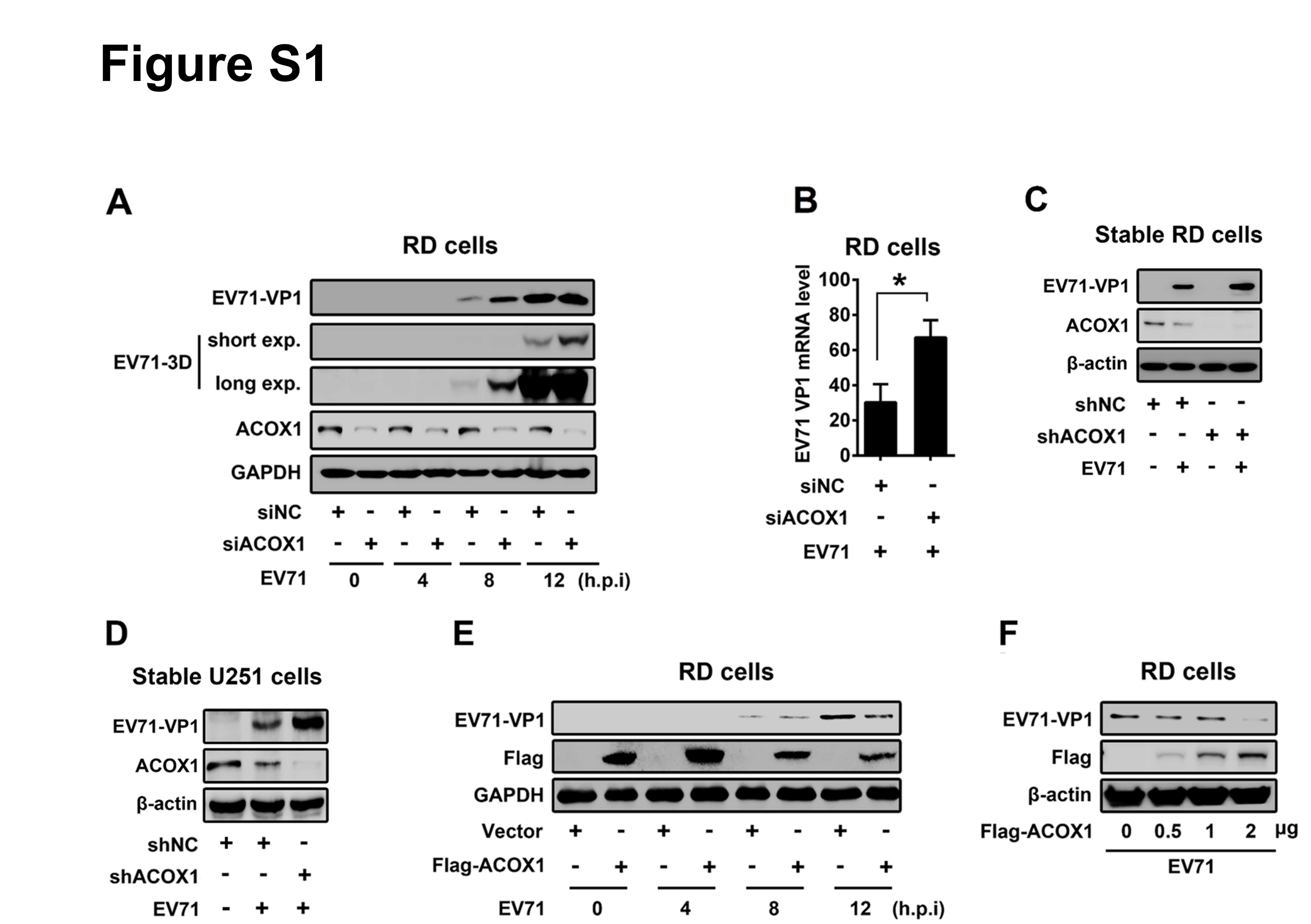

Supplement: Supplemental Material [file kvir-11-01-1766790-s001.tif]
